# RESTING STATE ELECTROENCEPHALOGRAPHY AND INHIBITORY CONTROL IN COGNITIVELY UNIMPAIRED OLDER ADULTS

**DOI:** 10.1093/geroni/igae098.2307

**Published:** 2024-12-31

**Authors:** Teresa Warren, Jaya Ashrafi, Shraddha Shende, Elizabeth Lydon, Raksha Mudar

**Affiliations:** University of Illinois Urbana-Champaign, Champaign, Illinois, United States; University of Illinois Urbana-Champaign, Champaign, Illinois, United States; Illinois State University, Normal, Illinois, United States; University of Illinois Urbana-Champaign, Champaign, Illinois, United States; University of Illinois, Champaign, Illinois, United States

## Abstract

Resting state electroencephalography (EEG) activity captures spontaneous neural activity while at rest. Mounting evidence suggests that resting state EEG activity is linked to cognitive performance and event-related potentials in young adults. However, little is known on the relationship between resting state EEG activity and event-related spectral perturbations (ERSPs) and/or behavioral performance linked to inhibitory control in cognitively unimpaired older adults (OA). This study examined the relationship between resting state alpha power and 1) alpha-band ERSPs and 2) behavioral measures during a Go/NoGo inhibitory control task in 22 OA (Age: M = 64.04, SD = 6.18 years; Montreal Cognitive Assessment score: M = 28.43, SD = 0.99). Participants’ EEG data were collected during eyes-open resting state and a Go/NoGo task. Fourier transformation-based spectral analysis was used to calculate power in the alpha frequency band (8-12 Hz), with absolute power spectral density for the resting state condition and event-related desynchronization (ERD) for the Go/NoGo task. Behavioral performance was assessed using reaction time on Go trials (M = 340.27 ms, SD = 33.71) and accuracy on Go (M = 0.92, SD = 0.13) and NoGo trials (M = 0.92, SD = 0.06). Based on preliminary analyses, lower absolute alpha power in resting state was correlated with better accuracy (r(4) = -0.995, p =.005) and lower alpha ERD (r(4) = -0.96, p =.005) on Go trials. These preliminary results suggest that resting state brain oscillations may predict brain dynamics and performance linked to inhibitory control in OA.

